# Digital and analogue modulation and demodulation scheme using vortex-based spin torque nano-oscillators

**DOI:** 10.1038/s41598-020-68001-6

**Published:** 2020-07-07

**Authors:** Alex S. Jenkins, Lara San Emeterio Alvarez, Paulo P. Freitas, Ricardo Ferreira

**Affiliations:** International Iberian Nanotechnology Laboratory, INL, Av. Mestre José Veiga s/n, 4715-330 Braga, Portugal

**Keywords:** Spintronics, Electronic and spintronic devices

## Abstract

In conventional communications systems, information is transmitted by modulating the frequency, amplitude or phase of the carrier signal, which often occurs in a binary fashion over a very narrow bandwidth. Recently, ultra-wideband signal transmission has gained interest for local communications in technologies such as autonomous local sensor networks and on-chip communications, which presents a challenge for conventional electronics. Spin-torque nano-oscillators (STNOs) have been studied as a potentially low power highly tunable frequency source, and in this report we expand on this to show how a specific dynamic phase present in vortex-based STNOs makes them also well suited as Wideband Analogue Dynamic Sensors (WADS). This multi-functionality of the STNOs is the basis of a new modulation and demodulation scheme, where nominally identical devices can be used to transmit information in both a digital or analogue manner, with the potential to allow the highly efficient transmittance of data.

## Introduction

Spin torque nano-oscillators (STNOs) are nanoscale tunable multifunctional radio-frequency devices which have been proposed for a diverse variety of applications^1^, ranging from wireless communications^2–6^, to nanoscale magnetic field detectors for magnetic hard drive read heads^7, 8^ and bio-sensors^9^ and more recently the building blocks of novel bio-inspired computing architectures for neuromorphic computing^10–14^.

With the emergence of the Internet of Things and the realisation of smart industries, cities etc., more and more data is being transmitted, and new architectures are required to allow a more efficient transfer of information. STNOs have been proposed for a range of different components of high frequency technologies, from rf signal generation^15–24^ and detection^25–29^, to rectification^30,31^ and mixing^32–34^. STNOs have many key strengths which include (i) multi-functionality^1^, (ii) scalability^35^, (iii) tunability^15–17^, (iv) coupling (either via dipolar coupling^36^, spin waves^37^ or electrical^38–41^) and (v) capacity for integration with CMOS technologies.

An STNO usually consists of a magnetic tunnel junction (MTJ), with free and fixed magnetic layers separated by an insulating layer, and where the free layer oscillates at high frequencies due to either a dc current, via the spin-transfer torque effect^42,43^, or a resonant radio-frequency current^30^ or magnetic field^44–46^. The magnetisation of the free layer impacts significantly the nature of the resultant dynamics, depending upon whether the free layer is homogeneously in-plane^42,43^ or non-homogenous (i.e. a magnetic vortex)^15–21^.

**Figure 1:**
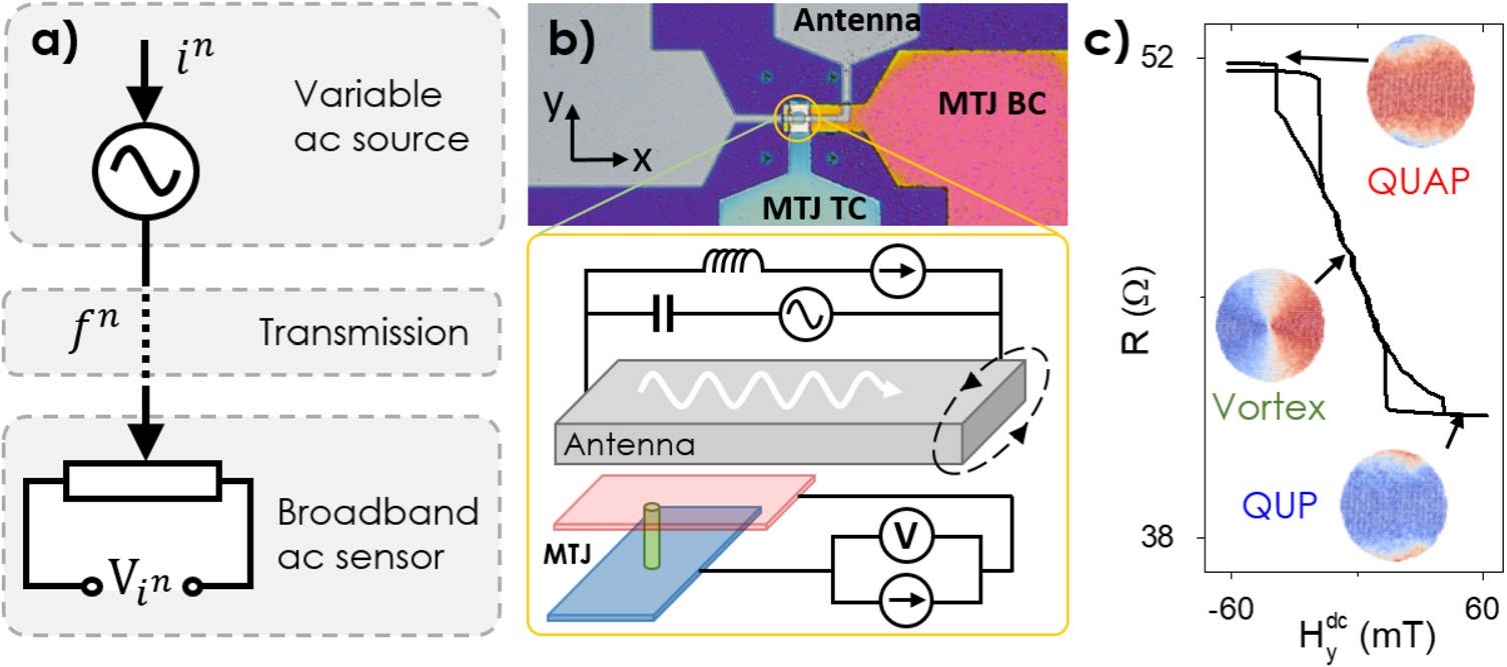
(**a**) Schematic representation of the modulation and demodulation scheme and (**b**) single device schematic and (**c**) transfer curve showing possible magnetisation states of the free layer, (insets are micromagnetic simulations).

In this report we propose a fully spintronic modulation and demodulation scheme, with two nominally identical STNOs, one of which is operating as a variable ac source, which can modulate the information to *n* possible frequency states by tuning an external parameter *i*, and the other of which is a broadband frequency to voltage converter which is capable of demodulating the signal, shown schematically in Fig. 1a. The maximum data rate demonstrated for spin-torque induced binary frequency shift keying is limited by the relaxation rate of the STNO^32^, and around ~ 1 MHz for a vortex based STNO^47^, but it has been proposed that field modulation could overcome this^48^.

The modulation and demodulation scheme presented in Fig. 1 has the potential to radically increase the efficiency of the transfer of data by utilising a new communications paradigm where information is transferred over a truly wideband frequency range. Whilst this type of communications paradigm might be impractical for wireless communications which are heavily restricted to relatively narrow bands (i.e. mobile phones, wifi etc.) emerging local autonomous sensors and on-chip communications applications, which require only very local data communication, are less restricted and would benefit from the increased data transmission efficiency and multiplexing.

As STNOs are inherently analogue devices, there are two possibilities for this modulation and demodulation transmission scheme: digital or analogue. If the external input parameter, *i*, has M discrete values, then an alphabet of M characters can be digitally sent via the carrier frequency, known as M-ary shift keying (i.e. if M = 2 then the system is binary). However, conversely, the input parameter can be varied in a continuous manner, and information can be modulated in an analogue fashion onto the carrier signal.

There are distinct advantages to both digital and analogue modulation and the final choice of which scheme is more relevant depends largely on the targeted application. Analogue modulation makes good use of bandwidth and can efficiently transmit information accurately, but is also in general less tolerant of noise. Digital modulation requires some level of synchronisation and is less efficient, however is in general more tolerant to low levels of noise and is more compatible with existing wireless technologies.

## Methods

In Fig. 1b, a schematic of a single device is presented, where an MTJ has been fabricated in the shape of a circular nanopillar with diameter d = 600 nm which is electrically connected with top and bottom electrodes and has a stack consisting of 5 Ta/50 CuN/5 Ta/50 CuN/5 Ta/5 Ru/6 IrMn/2.0 CoFe_30_/0.7 Ru/2.6 Co_40_Fe_40_B_20_/MgO/2.0 Co_40_Fe_40_B_20_/0.2 Ta/7.0 NiFe/10 Ta/7 Ru (thicknesses in nanometers), and has a microstrip antenna 600 nm directly above. The devices are identical to those presented previously in refs.^28,45^. The MgO layer was deposited as a wedge with variable thickness resulting in an RxA distribution between 1 and 30 Ωµm^2^. Similar trends were observed for all values of RxA. The magnetisation of the reference layer (2.6 nm Co_40_Fe_40_B_20_) is in-plane and remains fixed, and the free layer (2.0 Co_40_Fe_40_B_20_/0.2 Ta/7.0 NiFe) is initially in a vortex ground state, but can be switched to an in-plane state via a resonant excitation^19^ or static in-plane field (Fig. 1c). After switching occurs the magnetisation is in a C-state (see micromagnetic simulation as insets in Fig. 1c), where the majority of the magnetisation is aligned either parallel or anti-parallel to the fixed layer, and is referred to as either the quasi-uniform parallel (QUP) or antiparallel (QUAP) state, which depends on the sign of any in-plane magnetic fields present. A current passing across the antenna will produce a predominantly in-plane magnetic field across the MTJ collinear with the magnetisation direction of the reference layer. The fundamental resonant mode of a magnetic vortex is the gyrotropic mode and corresponds to an orbital motion of the vortex core around some fixed point, with mode frequencies typically 100 MHz–1 GHz^16^.

**Figure 2:**
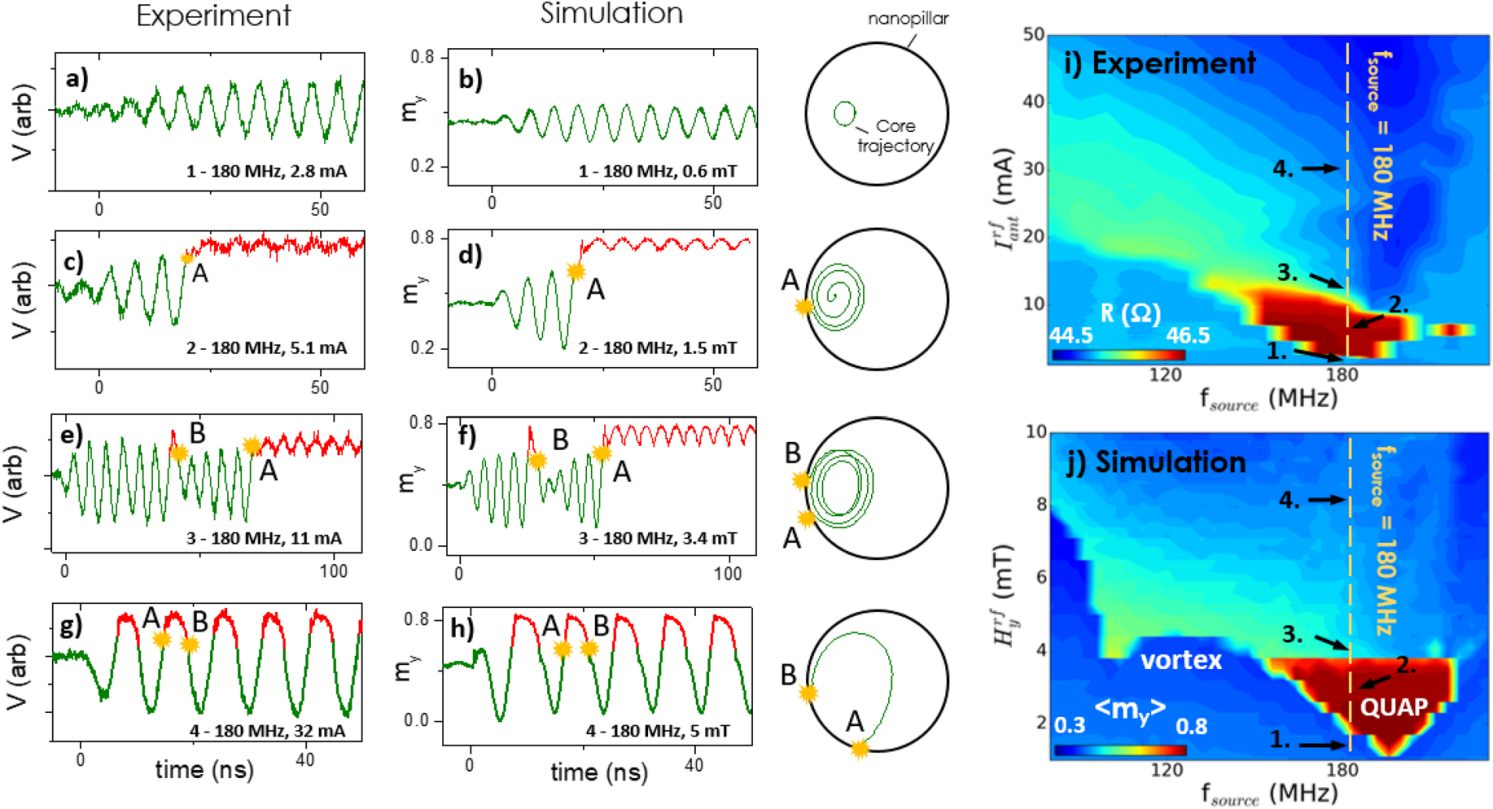
Voltage measured experimentally (**a**,**c**,**e**,**g**) and the magnetisation component collinear to the reference layer (m_y_) from micromagnetic simulations (**b**,**d**,**f**,**h**) as a function of time for different excitation strengths. Typical trajectories of the vortex core calculated from the micromagnetic simulations are presented, where A and B are the point of core expulsion and renucleation, respectively. (**i**) Experimental and (**j**) micromagnetic dynamical state diagram where (**i**) the resistance and (**j**) the average magnetisation < m_y_ > are plotted as a function of the excitation strength (${I}_{ant}^{rf}$/${H}_{y}^{rf}$) and excitation frequency.

**Figure 3:**
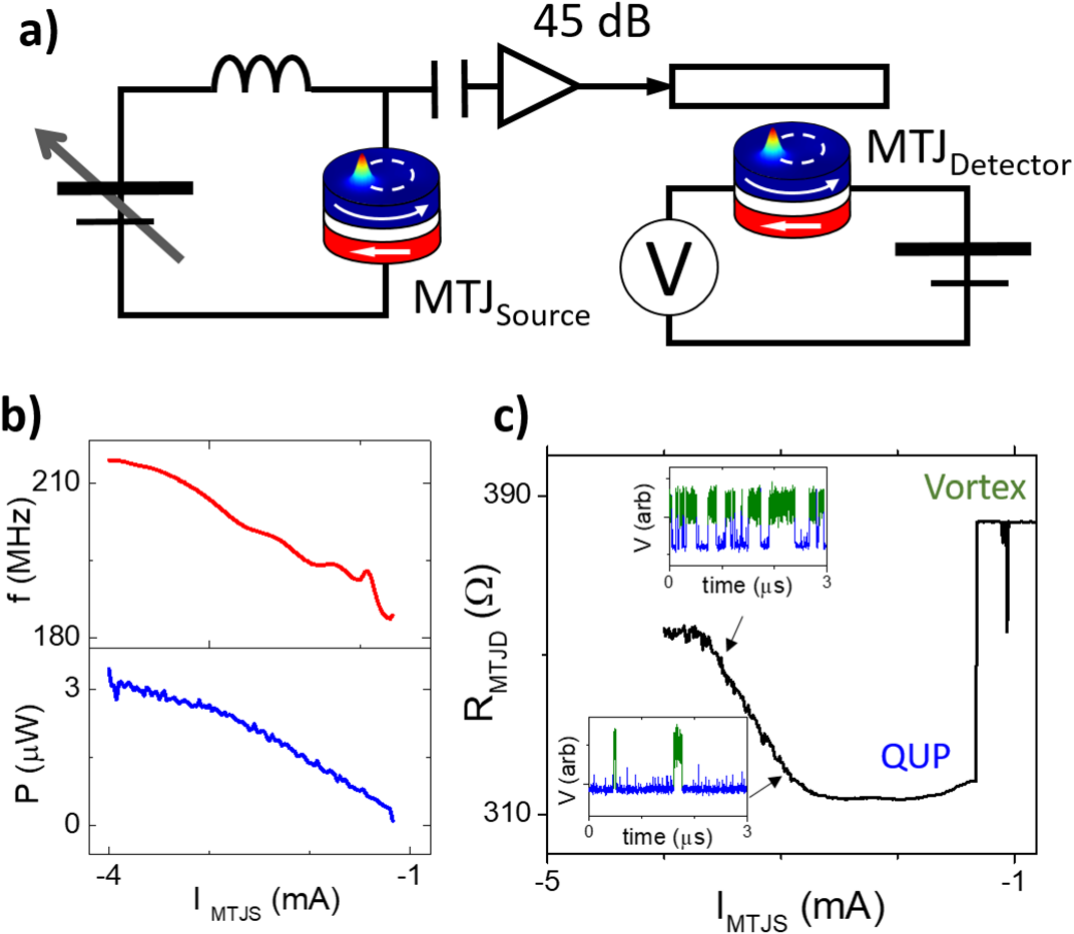
(**a**) schematic circuit showing how the signal created by MTJ_source_ is fed into the antenna of MTJ_detector_. The (**b**) spectral properties of MTJ_source_ alongside the c) resultant resistance response of MTJ_detector_ as a function of the current applied to the MTJ_source_ (I_MTJS_). The insets in (**c**) show example time traces, where the free layer is transitioning between the vortex (green) and QUP (blue) states.

**Figure 4:**
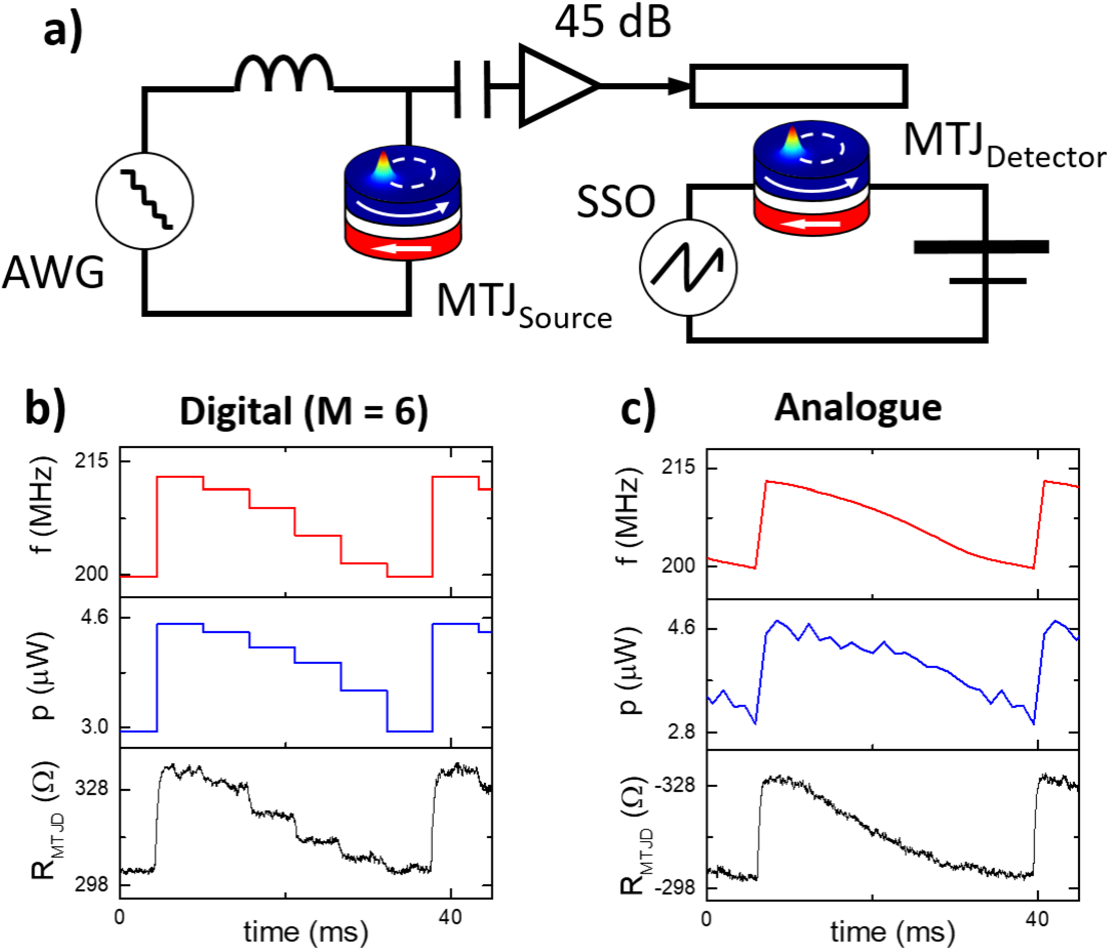
(**a**) STNO based modulation and demodulation scheme with (**b**) six discrete dynamic states and (**c**) analogue variation of frequency and power and the resultant resistance measured across the MTJ_detector_ as a function of time.

For the micromagnetic simulations, the static in-plane field produced by the antenna is reproduced with a simple in-plane field, ${\text{H}}_{\text{y}}^{\text{d}\text{c}}$. The result of the static in-plane field is to displace the vortex core around 30% of the diameter towards the edge of the nanopillar in the x-direction. In Fig. 2 an additional perpendicular field, H_z_ = 0.4 T is also applied, which tilts the magnetisation out-of-plane and acts to reduce the energy barrier between the two magnetisation states and promotes a more non-hysteretic type of switching, discussed further in Supplementary Fig. SI1 in the supplementary information. It is important to note that in Figs. 3 and 4 there is a discussion of potential applications, so these experiments were carried out in the absence of the perpendicular field, as this is an important challenge to be overcome for realistic device integration.

## Results

When a radio-frequency signal arrives to the integrated field line antenna, it will result in a dynamic response from the free layer of the MTJ, the nature of which depends on the frequency and power of the incoming signal. In Fig. 2a–h, the dynamic response of the free layer is summarised both via experimental measurement of the voltage in the time domain under the application of a constant dc current (Fig. 2a,c,e,g), and via the component of the magnetisation collinear with the applied magnetic field, m_y_, found with micromagnetic simulations^49^ (Fig. 2b,d,f,h). The material parameters used for the simulations were M_sat_ = 720 × 10^3^ A/m, A_ex_ = 1.2 × 10^–11^ Jm^−1^, alpha = 0.01 and T = 300 K similar to ref^28^. The trajectory of the vortex core calculated from the micromagnetic simulations is presented on the far right of the figure. The voltage is measured with ${\text{I}}_{\text{M}\text{T}\text{J}}^{\text{d}\text{c}}$ = 8 mA which was below the critical current necessary to observe steady state spin torque induced oscillations in this device, which are not observed in d = 600 nm devices before breakdown current is reached around 10 mA.

In all the data shown in Fig. 2a–h, the rf current is switched on at t = 0, and the frequency of the rf current is 180 MHz and the excitation strength (i.e. the amplitude of the radio frequency current experimentally applied across the antenna, ${\text{I}}_{\text{a}\text{n}\text{t}}^{\text{r}\text{f}}$, or the radio frequency magnetic field applied in the magnetic simulations, ${\text{H}}_{\text{y}}^{\text{r}\text{f}}$) is selected to demonstrate different dynamic behaviours. A dc current of ${\text{I}}_{\text{a}\text{n}\text{t}}^{\text{d}\text{c}}$ = − 50 mA was applied to the antenna during the measurement which was reproduced via an in-plane field of ${\text{H}}_{\text{y}}^{\text{d}\text{c}}$ =  − 8 mT for the micromagnetic simulations. There is qualitatively a close agreement between the experimental results and the simulations about the behaviour of the magnetisation of the free layer.

For the relatively low excitation (i.e. Fig. 2a,b, ${\text{I}}_{\text{a}\text{n}\text{t}}^{\text{r}\text{f}}$ = 2.8 mA, ${\text{H}}_{\text{y}}^{\text{r}\text{f}}$ = 0.6 mT), the radio frequency in-plane magnetic field results in the vortex core gyrating with a circular trajectory which is relatively constant as a function of time. The trajectory of the vortex core, calculated by simulations and shown on the right, shows a roughly circular orbit which is offset due to the presence of a constant in-plane field, ${\text{H}}_{\text{y}}^{\text{d}\text{c}}$ =  − 8 mT. As the excitation strength is increased (i.e. Fig. 2c,d), ${\text{I}}_{\text{a}\text{n}\text{t}}^{\text{r}\text{f}}$ = 5.1 mA, ${\text{H}}_{\text{y}}^{\text{d}\text{c}}$ = 1.5 mT), the vortex core is excited to larger orbits which approach the edge of the nanopillar and results in the expulsion of the vortex core and the free layer enters the quasi-uniform anti-parallel (QUAP) magnetisation state (position A), as discussed previously in ref.^19^. At these low excitation strengths, this effect is strongly hysteretic and after the vortex expulsion, the free layer remains stable in the QUAP state.

As the excitation strength is further increased (i.e. Fig. 2e,f, ${\text{I}}_{\text{a}\text{n}\text{t}}^{\text{r}\text{f}}$ = 11 mA, ${\text{H}}_{\text{y}}^{\text{r}\text{f}}$ = 3.4 mT), however, the QUAP state becomes destabilised by the large radio frequency magnetic field and the free layer starts to switch back and forth in a probabilistic manner (i.e. non-periodic) between the two magnetisation states as a function of time. When the vortex core is renucleated (position B) the orbit is initially destabilised but gradually stabilises with time before the vortex core reaches the edge of the nanopillar and is re-expelled (position A), and this cycle continues with time in a relatively stochastic manner, switching back and forth between the two states.

When the excitation strength is increased even more (i.e. Fig. 2g) and h, ${\text{I}}_{\text{a}\text{n}\text{t}}^{\text{r}\text{f}}$ = 32 mA, ${\text{H}}_{\text{y}}^{\text{r}\text{f}}$ = 5 mT) the in-plane magnetic field starts to strongly destabilise both magnetic states, which results in the free layer starting to continuously switch back and forth between the vortex state and the QUAP state in a periodic manner, where the vortex core is renucleated (position B) and has time for one full gyration, before being re-expelled (position A).

In Fig. 2i,j we expand on the frequency and power dependence of the MTJ by presenting the dynamical state diagram. The dynamical state diagram is presented experimentally by measuring the average resistance (i.e. R_avg_) and also with micromagnetic simulations, where the average magnetisation in the y direction is plotted, < m_y_ > . A good qualitative agreement between the experimental data and the simulations can be seen in terms of the system behaviour. The positions 1–4 are labelled, indicating the regions of different dynamic behaviour as a function of incoming frequency and power (i.e. 1—resonant gyrotropic core motion, 2—hysteretic core expulsion, 3—non-periodic core expulsion and renucleation and 4—periodic core expulsion and renucleation).

The free layer is initially in the vortex state and an rf signal is passed across the antenna at a fixed frequency, and the strength of the signal is swept from low to high. The first obvious feature of the dynamical state diagram is the transition from the vortex state to the QUAP state (i.e. red region). This hysteretic transition, corresponding to the behaviour discussed in Fig. 2c,d, occurs around the gyrotropic frequency of the vortex (i.e. 180 MHz).

As the power is increased (${\text{I}}_{\text{a}\text{n}\text{t}}^{\text{r}\text{f}}$ > 11 mA and ${\text{H}}_{\text{y}}^{\text{r}\text{f}}$ > 3.4mT) the free layer enters a region of continuous state transitions between the vortex state and the QUAP state characterised by the vortex core expulsion and renucleation discussed in Fig. 2e–h. Within this region of continual state transitions, the amount of time the free layer spends in either the QUAP state or the vortex state is strongly dependent on the excitation signal. In this region, the time averaged magnetisation, and therefore the resistance via the tunnelling magnetoresistance effect, is strongly dependent on the frequency and amplitude of the excitation signal. If over a certain frequency or power range, it is possible to find a one-to-one relation in the resistance for a specific frequency or power, this can be used to identify the incoming dynamic state of a signal (i.e. x(f,p)).

In Fig. 3 we exploit this one to one relation to demonstrate how a vortex-based STNO can be used a wideband analogue dynamic sensor (WADS). Instead of using an rf source as in Fig. 2. In Fig. 3 we use two nominally identical vortex STNOs (d = 300 nm), but where one acts as a highly tunable high frequency source (MTJ_source_) and another which acts as wideband analogue dynamic sensor (MTJ_detector_). A variable dc current is applied to MTJ_source_, which generates a tunable high frequency signal which is amplified and passed across the integrated field line antenna above MTJ_detector_, shown schematically in Fig. 3a.

In Fig. 3b, the dc current dependence of the MTJ_source_ (i.e. I_MTJS_), which has been characterised with a spectrum analyser, is shown and an rf signal is seen to be generated with a frequency which increases from 180 to 210 MHz as a function of I_MTJS_, with a Q factor (f/Δf) of around 4,000. The power increases relatively steadily above the critical current (I_MTJS_ = 1.1 mA), reaching around P = 3 µW. In Fig. 3c, the resistance of the second MTJ which is acting as the signal detector (R_MTJD_) is measured as a function of the current applied to the first MTJ acting as the source (I_MTJS_). The free layer is initially in the vortex state, and when the current in MTJ_source_ is increased there is an initial decrease in resistance as the vortex is expelled and the free layer enters the quasi-uniform parallel (QUP) state (note: the free layer enters either the QUAP or QUP state depending solely on the direction of the dc field produced by the field line antenna, which in this case is positive). As I_MTJS_ is further increased, the current in the MTJ_source_ reaches a value of around I_MTJS_ =  − 2.8 mA and the resistance of the detector starts to increase with a quasi-linear dependence until it saturates around I_MTJS_ =  − 3.7 mA.

The insets in Fig. 3c show the voltage of MTJ_detector_ measured in the time domain at two current values, and show that the free layer of MTJ_detector_ is constantly transitioning between two magnetic states, the vortex state (green) and the quasi-uniform parallel (QUP) state (blue), similar to that discussed in Fig. 2e,f. The average resistance of MTJ_detector_ depends on the relative amount of time the free layer spends in either magnetisation state, and as the frequency and power of the signal generated by MTJ_source_ increase, there is a corresponding change in the dynamic behaviour of the free layer of MTJ_detector_. Initially the free layer starts to transition between the QUP state and the vortex state infrequently, and as such the average resistance is closer to the QUP state. As I_MTJS_ is further increased, however, the number of transitions between the QUP and vortex states starts to increase and the average resistance increases quasi-linearly.

In Fig. 3 we demonstrated how MTJ_detector_ was sensitive to the signal generated by MTJ_source_, and in Fig. 4 we advance this by demonstrating a modulation and demodulation scheme which uses nominally identical magnetic tunnel junctions as both the modulator and demodulator. An important aspect of many wireless communications systems is shift-keying, where information can be encoded into the frequency, amplitude or phase of a radio frequency signal^50,51^. Shift-keying has been demonstrated previously with STNOs^3,4^ but without an integrated means of demodulating the signal and thus de-encoding the information. In Fig. 4a we present the schematic of the circuit used for the modulation and demodulation scheme. An arbitrary waveform generator (AWG) can apply either distinct digital voltages or a ramped continuous variation of the voltage to the MTJ_source_ over a fixed period of time. This results in either digital or analogue encoding of the information to the carrier signal. This signal is amplified and sent to the field line antenna of an adjacent and nominally identical nanodevice which demodulates the data. The voltages applied by the arbitrary waveform generator are quasi-dc so that they would be filtered by the bias tee, and not be sent to the MTJ_detector_. There is significant perspective for improvement for both MTJ_detector_ and MTJ_source_, in terms noise and modulation and demodulation speed in order to increase the relative data rate.

In Fig. 4b, the arbitrary waveform generator applies six distinct voltages (i.e. M = 6), from − 340 to − 420 mV in steps of 16 mV (− 1.7 to − 2.1 mA in steps of 0.08 mA), to the MTJ_source_ over a fixed period of time, which results in six different dynamic states, x_i_(f_i_,p_i_) with different frequencies and powers being passed to the antenna of MTJ_detector_. In Fig. 4b), the frequency and power of the six distinct dynamic states are shown as a function of time, and the resistance response of the MTJ_detector_ can be seen to display discrete steps, corresponding to each dynamic state produced by MTJ_source_. With respect to the transmission scheme discussed in Fig. 1a, this corresponds to M = 6 inputs, ${i}^{1-6}$, resulting in M = 6 outputs ${V}_{{i}^{1-6}}$.

In Fig. 4c, the voltage applied to MTJ_source_ is ramped continuously resulting in an analogue change in the frequency and power. Due to the analogue frequency and power dependence of the resistance of MTJ_detector_, discussed in Fig. 3, there is a resultant analogue demodulation of the information.

The effective data rate in Fig. 4 will only be of the order of 100–1,000 bits/s, which is very low compared to current state of the art, however this relatively low number is due to the imperfect experimental set-up and can be significantly enhanced with more integrated and customised circuit designs. It is important to note that the power consumption in the MTJ will be around 1–10 µW, relative to the 0.1–1 W necessary for the amplifier. As a future perspective, a single MTJ which can create 256 dynamic states at a rate of 10 MHz would be capable of a potential maximum data transfer energy efficiency of E = 8 × 10^14^ bit/J, compared to current state of the art around 10 × 10^7^ bit/Joule^52^ , however for this type of transmission scheme to become viable the main focus must be on creating optimised electrical rf circuitry.

## Conclusion

In conclusion, we explore the magnetisation dynamics generated in vortex-based STNOs when excited by large radio-frequency magnetic fields, and show how this makes them exciting candidates as wideband analogue dynamic sensors. Furthermore, we demonstrate an STNO-based digital and analogue modulation and demodulation transmission scheme using two nanoscale nominally identical magnetic tunnel junctions, with one acting as a highly tunable frequency source and the other as a wideband analogue dynamic sensor.

## Supplementary information

Supplementary information

## Data Availability

The data that support the findings of this study are available from the corresponding author upon request.

## References

[1] Locatelli N, Cros V, Grollier J (2014). Spin-torque building blocks. Nat. Mater..

[2] Choi HS (2015). Spin nano–oscillator–based wireless communication. Sci. Rep..

[3] Manfrini M (2011). Frequency shift keying in vortex-based spin torque oscillators. J. Appl. Phys..

[4] Ma, R. *et al.* Spin torque oscillator based BFSK modulation. in *2017 13th Conference on Ph.D. Research in Microelectronics and Electronics (PRIME)* 1–4 (IEEE, 2017). 10.1109/PRIME.2017.7974092.

[5] Ruiz-Calaforra A (2017). Frequency shift keying by current modulation in a MTJ-based STNO with high data rate. Appl. Phys. Lett..

[6] Sharma R (2015). Modulation rate study in a spin-torque oscillator-based wireless communication system. IEEE Trans. Magn..

[7] Sato R, Kudo K, Nagasawa T, Suto H, Mizushima K (2012). Simulations and experiments toward high-data-transfer-rate readers composed of a spin-torque oscillator. IEEE Trans. Magn..

[8] Braganca PM (2010). Nanoscale magnetic field detection using a spin torque oscillator. Nanotechnology.

[9] Fried JP, Metaxas PJ (2017). Nanoparticle-modified magnetic vortex dynamics. IEEE Magn. Lett..

[10] Grollier J, Querlioz D, Stiles MD (2016). Spintronic nanodevices for bioinspired computing. Proc. IEEE.

[11] Nikonov DE (2015). Coupled-oscillator associative memory array operation for pattern recognition. IEEE J. Explor. Solid-State Comput. Devices Circuits.

[12] Vodenicarevic D, Locatelli N, Abreu Araujo F, Grollier J, Querlioz D (2017). A nanotechnology-ready computing scheme based on a weakly coupled oscillator network. Sci. Rep..

[13] Torrejon J (2017). Neuromorphic computing with nanoscale spintronic oscillators. Nature.

[14] Romera M (2018). Vowel recognition with four coupled spin-torque nano-oscillators. Nature.

[15] Pribiag VS (2007). Magnetic vortex oscillator driven by d.c. spin-polarized current. Nat. Phys..

[16] Dussaux A (2010). Large microwave generation from current-driven magnetic vortex oscillators in magnetic tunnel junctions. Nat. Commun..

[17] Dussaux A (2014). Large amplitude spin torque vortex oscillations at zero external field using a perpendicular spin polarizer. Appl. Phys. Lett..

[18] Jenkins AS (2014). Controlling the chirality and polarity of vortices in magnetic tunnel junctions. Appl. Phys. Lett..

[19] Jenkins AS (2016). Spin-torque resonant expulsion of the vortex core for an efficient radiofrequency detection scheme. Nat. Nanotechnol..

[20] Tsunegi S (2014). High emission power and Q factor in spin torque vortex oscillator consisting of FeB free layer. Appl. Phys. Express.

[21] Tsunegi S (2018). Scaling up electrically synchronized spin torque oscillator networks. Sci. Rep..

[22] Grimaldi E (2014). Response to noise of a vortex based spin transfer nano-oscillator. Phys. Rev. B.

[23] Costa JD (2017). High power and low critical current density spin transfer torque nano-oscillators using MgO barriers with intermediate thickness. Sci. Rep..

[24] Tarequzzaman M (2019). Spin torque nano-oscillator driven by combined spin injection from tunneling and spin Hall current. Commun. Phys..

[25] Fang B (2016). Giant spin-torque diode sensitivity in the absence of bias magnetic field. Nat. Commun..

[26] Louis S (2018). Ultra-fast wide band spectrum analyzer based on a rapidly tuned spin-torque nano-oscillator. Appl. Phys. Lett..

[27] Miwa S (2014). Highly sensitive nanoscale spin-torque diode. Nat. Mater..

[28] Jenkins AS (2020). Wideband high-resolution frequency-to-resistance converter based on nonhomogeneous magnetic-state transitions. Phys. Rev. Appl..

[29] Menshawy S (2017). Spin transfer driven resonant expulsion of a magnetic vortex core for efficient rf detector. AIP Adv..

[30] Tulapurkar AA (2005). Spin-torque diode effect in magnetic tunnel junctions. Nature.

[31] Tarequzzaman M (2018). Broadband voltage rectifier induced by linear bias dependence in CoFeB/MgO magnetic tunnel junctions. Appl. Phys. Lett..

[32] Quinsat M (2014). Modulation bandwidth of spin torque oscillators under current modulation. Appl. Phys. Lett..

[33] Muduli PK, Pogoryelov Y, Mancoff F, Akerman J (2011). Modulation of individual and mutually synchronized nanocontact-based spin torque oscillators. IEEE Trans. Magn..

[34] Pufall MR, Rippard WH, Kaka S, Silva TJ, Russek SE (2005). Frequency modulation of spin-transfer oscillators. Appl. Phys. Lett..

[35] Chao X, Jamali M, Wang JP (2017). Scaling effect of spin-torque nano-oscillators. AIP Adv..

[36] Locatelli N (2015). Efficient synchronization of dipolarly coupled vortex-based spin transfer nano-oscillators. Sci. Rep..

[37] Houshang A (2016). Spin-wave-beam driven synchronization of nanocontact spin-torque oscillators. Nat. Nanotechnol..

[38] Lebrun R (2017). Mutual synchronization of spin torque nano-oscillators through a long-range and tunable electrical coupling scheme. Nat. Commun..

[39] Kaka S (2005). Mutual phase-locking of microwave spin torque nano-oscillators. Nature.

[40] Grollier, J., Boulle, O., Cros, V. & Fert, A. Synchronization of spin-transfer oscillators driven by stimulated microwave currents. in *INTERMAG 2006—IEEE International Magnetics Conference* 264–264 (IEEE, 2006). 10.1109/INTMAG.2006.375846.

[41] Singh H (2019). Mutual synchronization of spin-torque nano-oscillators via oersted magnetic fields created by waveguides. Phys. Rev. Appl..

[42] Kiselev SI (2003). Microwave oscillations of a nanomagnet driven by a spin-polarized current. Nature.

[43] Sankey JC (2008). Measurement of the spin-transfer-torque vector in magnetic tunnel junctions. Nat. Phys..

[44] Urazhdin S, Tiberkevich V, Slavin A (2010). Parametric excitation of a magnetic nanocontact by a microwave field. Phys. Rev. Lett..

[45] Jenkins AS, Alvarez LSE, Freitas PP, Ferreira R (2019). Nanoscale true random bit generator based on magnetic state transitions in magnetic tunnel junctions. Sci. Rep..

[46] Singh H (2017). Integer, fractional, and sideband injection locking of a spintronic feedback nano-oscillator to a microwave signal. Phys. Rev. Appl..

[47] Grimaldi, E. *et al.* Spintronic nano-oscillators: Towards nanoscale and tunable frequency devices. in *IFCS 2014–2014 IEEE International Frequency Control Symposium, Proceedings* (IEEE Computer Society, 2014). 10.1109/FCS.2014.6859850

[48] Purbawati A, Garcia-Sanchez F, Buda-Prejbeanu LD, Ebels U (2016). Enhanced modulation rates via field modulation in spin torque nano-oscillators. Appl. Phys. Lett..

[49] Vansteenkiste A (2014). The design and verification of MuMax3. AIP Adv..

[50] Muduli PK (2010). Nonlinear frequency and amplitude modulation of a nanocontact-based spin-torque oscillator. Phys. Rev. B Condens. Matter Mater. Phys..

[51] Sharma R (2017). A high-speed single sideband generator using a magnetic tunnel junction spin torque nano-oscillator. Sci. Rep..

[52] Bjornson, E. & Larsson, E. G. How Energy-Efficient Can a Wireless Communication System Become? in *Conference Record—Asilomar Conference on Signals, Systems and Computers* 2018-October, 1252–1256 (IEEE Computer Society, 2019).

