# Supplementary material for: Digital and analogue modulation and demodulation scheme using vortex-based spin torque nano-oscillators
Source: Sci Rep. 2020 Jul 7;10:11181. doi: 10.1038/s41598-020-68001-6 (PMC7341870; doi:10.1038/s41598-020-68001-6)
Supplement: Supplementary file 1 — Supplementary information [file 41598_2020_68001_MOESM1_ESM.docx]

**Digital and analogue modulation and demodulation scheme using vortex-based spin torque nano-oscillators – Supplementary information**

*Alex S. Jenkins*^1^, Lara San Emeterio Alvarez^1^, Paulo P. Freitas^1^, Ricardo Ferreira^1^*

1- International Iberian Nanotechnology Laboratory, INL, Av. Mestre José Veiga s/n, 4715-330, Braga, Portugal.

*corresponding author: [alex.jenkins@inl.int](mailto:alex.jenkins@inl.int)

For the experimental data shown in Figure 2 of the main manuscript, a perpendicular field is applied. The effect of this perpendicular field is to allow the periodic transitions shown labelled 4. These transitions are due to the free layer constantly and periodically switching between the vortex and the QUAP state. The reason this perpendicular field is applied is because, as shown in Figure SI1, the relative energy landscape of the vortex and QUAP states can be altered by the perpendicular field. The difference between the total energy of either state, E_Tvort_ - E_TQUAP_, is reduced as the perpendicular field is applied. This is because the Zeeman energy becomes the dominant energy terms, and the demagnetizing energies become more equivalent as the magnetization starts to tilt out-of-plane. As the difference between the energy levels becomes reduced, the non-hysteretic switching between states becomes easier.

The energy barrier which separates the vortex state and the QUAP state for a given magnetic field can be thought of as relating mostly to the formation and destruction of the vortex core. This can be seen as the exchange difference between the two states, E_exvort_ – E_exQUAP_, where the vortex state has a large exchange energy related to the vortex core. As the perpendicular field is increased, this acts to tilt the magnetization out-of-plane and increase the core size, which reduces the exchange cost associated with the core, thus making it easier for the free layer to switch back and forth in a non-hysteretic manner.

Figure SI1 – Energy difference of the vortex and QUAP states at a constant in-plane field of 5mT as a function of the perpendicular magnetic field, as found by micromagnetic simulations.
